# Vacation in Egypt associated with Shiga toxin-producing *Escherichia coli* infection in children and adolescents, northern Italy, 2023

**DOI:** 10.2807/1560-7917.ES.2024.29.30.2400056

**Published:** 2024-07-25

**Authors:** Thomas Ria, Maria Cristina Mancuso, Laura Daprai, Maria Francesca Liporace, Alessandra Gazzola, Sara Arnaboldi, Federica Vianello, Mario Luini, Dario Consonni, Gianluigi Ardissino, on behalf of the ItalKid-HUS Network

**Affiliations:** 1 Centro per la Cura e lo Studio della Sindrome Emolitica Uremica, Fondazione IRCCS Ca’ Granda Ospedale Maggiore Policlinico, Milan, Italy; 2 SC Patologia Clinica, Laboratorio di Microbiologia, Fondazione IRCCS CA’ Granda, Ospedale Maggiore Policlinico, Milan, Italy; 3 Istituto Zooprofilattico Sperimentale della Lombardia e dell’Emilia-Romagna, Lodi, Italy; 4 Department of Food Safety, Istituto Zooprofilattico Sperimentale della Lombardia e dell'Emilia-Romagna “Bruno Ubertini” (IZSLER), Brescia, Italy; 5 Institute of Agricultural Biology and Biotechnology, National Research Council, Lodi, Italy; 6 Epidemiology Unit, Fondazione IRCCS Ca’ Granda Ospedale Maggiore Policlinico, Milan, Italy; 7 The members of the ItalKid-HUS Network are acknowledged at the end of the article

**Keywords:** Shiga toxin, haemolytic uremic syndrome, acute kidney injury, Egypt, Europe, Italy, food-borne infections, waterborne infections, Shiga toxin-producing E. coli, STEC

## Abstract

**Background:**

Haemolytic uremic syndrome (HUS) is a severe complication of infection with Shiga toxin-producing *Escherichia coli* (STEC). Although the reservoirs of STEC are known, the source of the infection of sporadic cases is often unknown. In 2023, we observed several cases of bloody diarrhoea with STEC infection in children and adolescents returning from vacations.

**Aim:**

We aimed to explore the association between travel and bloody diarrhoea with STEC infection in children and adolescents.

**Methods:**

We included all children and adolescents with bloody diarrhoea with STEC infection identified in 2023 by the ItalKid-HUS Network surveillance system in northern Italy. We interviewed children’s families and sent a questionnaire on recent travels abroad. The exposure time was between 3 days after arrival abroad and 5 days after return home. A self-controlled case series (SCCS) design was used in the analysis.

**Results:**

Of the 43 cases, 11 developed HUS. Twenty-three cases did not travel abroad, while 20 had travelled to several destinations. The incidence rate ratio (IRR) associated with travel to Egypt was 88.6 (95% confidence interval (CI): 17.0–462). Serotype analysis excluded the possibility of a single strain causing the infections. We did not find the source of the infections.

**Conclusion:**

There is an elevated risk of acquiring STEC infection with bloody diarrhoea and HUS associated with travel to Egypt. Specific investigations to identify the source are needed to implement effective preventive measures.

**Key public health message**

**What did you want to address in this study and why?**

Shiga toxin-producing *Escherichia coli* (STEC) are food-borne bacteria that can cause gastrointestinal infections in humans. Haemolytic uraemic syndrome (HUS), a severe condition affecting kidneys, may complicate STEC infection. Given that the source of the infection is often unclear, we explored the association with travel and STEC infections with bloody diarrhoea in children and adolescents in northern Italy.

**What have we learnt from this study?**

Travel to Egyptian vacation sites was associated with an 89-fold higher risk of acquiring STEC infection with bloody diarrhoea compared to children not travelling to Egypt.

**What are the implications of your findings for public health?**

Travellers should ensure eating thoroughly cooked meat, not drinking tap water or ice made from tap water or unpasteurised dairy products and trying to avoid swallowing water when swimming. To decrease the number of STEC-related illnesses, prevention measures should focus on improving the safety of foods and beverages.

## Introduction

Infection with Shiga toxin-producing *Escherichia coli* (STEC) is an endemo-epidemic zoonosis causing gastrointestinal disease, bloody diarrhoea in particular, in children and adults all over the world [1].

Haemolytic uraemic syndrome (HUS) is a severe and life-threatening thrombotic microangiopathy that develops as a complication in 12–23% of paediatric patients infected with STEC in northern Italy [2]. The estimated annual incidence in Italy is 6.3 cases per million children and adolescents [3].

Cattle are the main reservoir of STEC, and humans can become infected via ingestion of insufficiently cooked meat, dairy products or vegetables contaminated with the pathogen. Other sources of infection include swimming in contaminated water or direct or indirect contact with animal faeces. Person-to-person transmission is also well documented [4]. However, the source of the infection remains unknown for most cases [5,6].

Despite combined efforts (agricultural, veterinary, public health and food safety) to control STEC, the incidence of HUS caused by STEC (STEC-HUS) has not decreased [7]. In northern Italy, a surveillance system aiming at an early detection and management of STEC infection in children and adolescents was established in 2009 (ItalKid-HUS Network). Recently, we have observed several cases of STEC infection or STEC-HUS in individuals returning from holidays abroad, thus we decided to investigate the association between travel and the risk of STEC infection in children and adolescents in northern Italy.

## Methods

### Surveillance and case definitions

We included all children and adolescents (< 18 years) with bloody diarrhoea with STEC infection identified by the ItalKid-HUS Network between 1 January and 31 December 2023, hereinafter called cases. The ItalKid-HUS Network is a surveillance system that screens for Shiga toxin genes from samples from children and adolescents with bloody diarrhoea. The network includes 63 paediatric units in northern Italy, with a referral paediatric population of 2.3 million (detailed methodological description of the ItalKid-HUS Network is provided elsewhere [5]).

We defined bloody diarrhoea as an acute (duration < 10 days) diarrhoea with visible blood in at least one bowel movement either seen by healthcare professionals or reported by caregivers.

Haemolytic uraemic syndrome is diagnosed as the concomitant presence of platelet consumption (platelet count < 150,000/mm^3^ or more than 50% acute reduction of platelet count), non–immune-mediated (Coombs negative) haemolysis (anaemia or undetectable haptoglobin or lactate dehydrogenase above age-specific upper limit of normal) and kidney injury (serum creatinine above the upper normal limit for age and sex or proteinuria or haematuria) in a patient with evidence of STEC infection (Shiga toxin genes detected from stool sample and/or anti-lipopolysaccharide positivity) [5].

### Microbiological methods

Shiga toxin genes were analysed from faecal samples by extracting DNA using the STARMag 96 X 4 Universal Cartridge Kit (Seegene Inc., South Korea) following the manufacturer’s instructions. The DNA was amplified by real-time PCR targeting the *stx1*, *stx2* and *eae* genes (Allplex Gastrointestinal Panel Assays, Seegeene and RealStar EHEC PCR Kit 2.0, Altona Diagnostics GmbH, Germany), along with the genes associated with the main serotypes causing HUS in Europe (O157, O26, O103, O111, O145 and O104), according to the methods recommended by the European Union Reference Laboratory (EURL) for *Escherichia coli* (Istituto Superiore di Sanità (ISS), Italy; https://www.iss.it/en/vtec-laboratory-methods). Bacteriological analyses were then performed on the samples positive for stx genes to isolate, when possible, the bacteria. The isolates were then sequenced on an Illumina MiSeq platform (Illumina, the Unites States (US)) using the Illumina MiSeq Reagent Kit v2 (500-cycle), the Illumina DNA Prep Kit and the Nextera DNA CD Index Kit. The reads were analysed with FastQC, Trimmomatic and SPAdes tools provided by the ARIES Public Galaxy Server (https://aries.iss.it).

### Epidemiological investigations

We contacted the families of the cases by phone and administered a questionnaire after an informed consent had been obtained. Information on travel destinations, dates of arrival and return during 2023, and the date of symptom onset were collected. We also collected information on exposures during travel, including the site of vacation, name of the holiday resort, food and beverages consumed, excursions outside the holiday resort and swimming.

A self-controlled case series (SCCS) design was used; this is a case-only study in which the incidence of a clinical event (outcome) occurred in a defined time interval after the exposure is compared with the incidence in referent (unexposed) periods before and after the exposed periods [8-10].

Interviews were administered during the study period and updated in January 2024 to cover the whole year (as required by the SCCS design).

For each case, person-days were calculated in various time windows before, during and after each travel abroad. Each case contributed with person-time to the entire 12-month period [11].

The country of infection was defined as the country where the patient most likely acquired the infection, considering the incubation time of STEC infection (2–5 days) and the subsequent development of symptoms. For each case and travel, we classified as exposed (i.e. at risk) the person-days as the day 3 after the arrival in the country to the day 5 after return to Italy. All remaining person-days (before travel, first and second day abroad and > 5 days after the return) were classified as unexposed. Since many of the cases had been in Egypt, we created an additional variable coded as follows: 0 (unexposed), 1 (exposed abroad, not in Egypt) and 2 (exposed in Egypt).

Using SCCS, comparisons are self-matched (within individual), thus fixed confounders (e.g. sex and age) are automatically adjusted. However, time-varying variables are not adjusted; therefore, since the risk of developing HUS varies during the year, a time-varying covariate was created by dividing the observation time to 12 months. Then we fitted conditional (fixed effect) Poisson regression models adjusted for month to calculate the incidence rate ratio (IRR) and the 95% confidence interval (CI) of exposed vs unexposed periods. One important assumption of the SCCS method is that occurrence of an event does not alter the probability of subsequent exposure. In this situation, a simple way to correct for this is to use a pre-exposure risk period to remove this time from the baseline [8-11]. It is possible that the attitude to travel of individual cases was affected by the infection (i.e. some families may have delayed travels after the STEC infection). In this case, the probability of exposure (travel) is temporally reduced after the event. For this reason, as suggested by the proponents of the SCCS method, in two sensitivity analyses we included in the Poisson models pre-exposure periods of 45 or 60 days before the first travel following STEC infection [8-11].

Statistical analyses were performed with Stata 18 (StataCorp LLC, US), using the commands stset, stsplit and xtpoisson.

## Results

In 2023, 43 cases (24 males) were identified, aged 0.7–17.3 years. Of these 43, 11 developed HUS. Each case contributed with person-time for the entire 12-month period, for a total of 15,695 (43 × 365) person-days.

Most cases (n = 31) occurred between May and September (4 in May, 4 in June, 4 in July, 12 in August and 7 in September). During the exposed periods, 12 cases (900 person-days) were observed, with a month-adjusted IRR of 17.2 (95% CI: 6.2–48.0). No effect modification by sex and age was observed (results not shown).

Most (n = 23) cases had not travelled abroad and were considered infected in Italy, 12 travelled to Egypt and 8 to other countries (Table 1).

**Table 1:** Clinical characteristics and travel information of children and adolescents with bloody diarrhoea and infected with Shiga toxin-producing *Escherichia coli,* northern Italy, 2023 (n  =  43)

ID	Sex	Age (years)	Month of symptom onset	Disease	Shiga toxin type	Serotype	Travel abroad (country)	Days abroad	Country of infection
1	F	11.5	January	Enteritis	1 and 2	O157	None	NA	Italy
2	M	0.8	February	Enteritis	2	O116	None		Italy
3	F	12.3	March	HUS	1 and 2	O157	None		Italy
4	M	7.5	March	Enteritis	1^a^	ND	None		Italy
5	M	11.0	March	Enteritis	2^a^	ND	None		Italy
6	M	1.0	April	Enteritis	1^a^	O151	None		Italy
7	F	3.4	May	Enteritis	1	O71	None		Italy
					2	O26			
8	M	3.7	May	HUS	1 and 2	O111	Egypt, Spain	14	Egypt
9	M	0.7	May	Enteritis	2^a^	ND	Egypt	70	Italy
10	M	6.0	May	Enteritis	1	O127	Romania, Türkiye	17	Italy
11	F	12.5	June	Enteritis	2^a^	O103 and O145^a^	None	NA	Italy
12	F	2.3	June	Enteritis	2^a^	Other^c^	None		Italy
13	F	0.9	June	Enteritis	2^a^	Other^c^	Croatia	8	Italy
14	M	1.3	June	Enteritis	1	O186	Egypt	9	Egypt
15	M	6.7	July	HUS	ND	ND	Egypt	8	Egypt
16	F	8.7	July	HUS	1 and 2	O71	Egypt	8	Egypt
17	M	13.3	July	HUS	1	O26	None	NA	Italy
18	M	3.3	July	Enteritis	2	O157	None		Italy
19	M	10.9	August	Enteritis	1	O26	France	4	Italy
20	F	1.1	August	Enteritis	1^a^	ND	Australia	290	Italy
21	M	15.8	August	Enteritis	2^a^	ND	None	NA	Italy
22	F	15.1	August	HUS	1 and 2^a^	O157^a^	Egypt	8	Egypt
23	F	9.3	August	HUS	1 and 2^a^	Other^c^	Egypt, Spain, Switzerland	15	Egypt
24	F	0.7	August	HUS	ND	ND	Kosovo^§^	18	Kosovo^§^
25	M	2.2	August	Enteritis	1	O111	Switzerland, Egypt	225	Egypt
26	M	2.6	August	Enteritis	1	O71	Egypt	14	Egypt
27	M	5.2	August	Enteritis	Stx^b^	ND	None	NA	Italy
28	F	4.9	August	HUS	2^a^	ND	None		Italy
29	M	1.4	August	Enteritis	1	O5	None		Italy
30	F	11.4	August	Enteritis	2^a^	ND	UK, Egypt	19	Italy
31	M	1.1	September	Enteritis	2	O26	Romania	29	Italy
32	M	17.3	September	Enteritis	1 and 2	O157	None	NA	Italy
33	M	10.0	September	Enteritis	1 and 2^a^	ND	Albania	25	Albania
34	F	7.5	September	Enteritis	1^a^	O111^a^	None	NA	Italy
35	F	1.2	September	Enteritis	1 and 2^a^	O157^a^	None		Italy
36	F	7.7	September	HUS	2^a^	Other^c^	None		Italy
37	M	15.9	September	Enteritis	2^a^	ND	None		Italy
38	F	1.7	November	Enteritis	1	O111	Spain, Egypt	16	Egypt
39	M	0.5	November	Enteritis	1^a^	Other^c^	Egypt	8	Egypt
40	M	3.8	November	Enteritis	1 and 2^a^	O157^a^	None	NA	Italy
41	F	2.2	November	Enteritis	1 and 2	O157	None		Italy
42	F	5.0	December	Enteritis	2^a^	Other^c^	None		Italy
43	M	2.4	December	HUS	2^a^	Other^c^	Spain	11	Italy

F: Female; HUS: haemolytic uraemic syndrome; ID: identification code; M: male, NA: not applicable; ND: not determined; UK: the United Kingdom.

^a^ Determined in stools by real-time PCR.

^b^ The test could not distinguish between Stx1 and Stx2.

^c^ Other serotype than O157, O26, O103, O111, O1O4 or O145.

We considered that 10 cases acquired the infection in Egypt, thus they were in the exposed window after travelling to Egypt (472 person-days). Seven acquired the disease while in Egypt, and three started presenting symptoms on the second day after their return to Italy. Five of these 10 cases subsequently developed HUS. The corresponding IRR was 88.6 (95% CI: 17.0–462). In the sensitivity analyses, in which we included pre-exposure periods of 45 and 60 days, the IRRs were higher (107 and 149, respectively).

Two exposed cases (428 person-days) developed the disease while travelling in other European countries than Italy, with no increased risk (IRR: 1.67; 95% CI: 0.26–10.7). The Shiga toxin type and the serotypes are shown in Table 1. We could not find any other exposures than travel to Egypt for the 10 cases visiting Egypt (Table 2).

**Table 2:** Children and adolescents with bloody diarrhoea and infected with Shiga toxin-producing *Escherichia coli*, by exposures related to travel to Egypt, northern Italy, 2023 (n  =  10)^a^

ID	Town	Holiday resort	Drinking water	Ice	Fresh fruit	Fresh vegetables	Meat	Dairy products	Street food	Swimming water
8	Sharm El-Sheikh	A1	Bottled and water dispensers	No	Yes	No	Yes	Yes	No	Pool and sea
14	Marsa Matruh	B1	Bottled	No	Yes	No	Yes	Yes	No	Pool
15	Marsa Matruh	B1	Bottled	Yes	Yes	No	Yes	Yes	No	Pool and sea
16	Marsa Alam	C1	Bottled	Yes	Yes	Yes	Yes	Yes	No	Pool and sea
22	Sharm El-Sheikh	A2	Bottled and water dispensers	No	No	No	No	No	No	Pool and sea
23	Marsa Alam	C2	Bottled	No	Yes	No	Yes	Yes	No	Pool and sea
25	Sharm El-Sheikh	A3	Bottled	No	Yes	No	No	Yes	No	Pool and sea
26	Hurgada	D1	Bottled	No	Yes	No	Yes	Yes	No	Pool and sea
38	Marsa Alam	C3	Bottled	No	Yes	Yes	Yes	Yes	No	Pool and sea
39	Marsa Alam	C4	Bottled	No	Yes	No	No	No	No	Pool and sea

ID: identification code.

^a^ Only cases considered infected in Egypt included in the table.

Geographic locations of holiday resorts of cases infected in Egypt are presented on a map (Figure).

**Figure:**
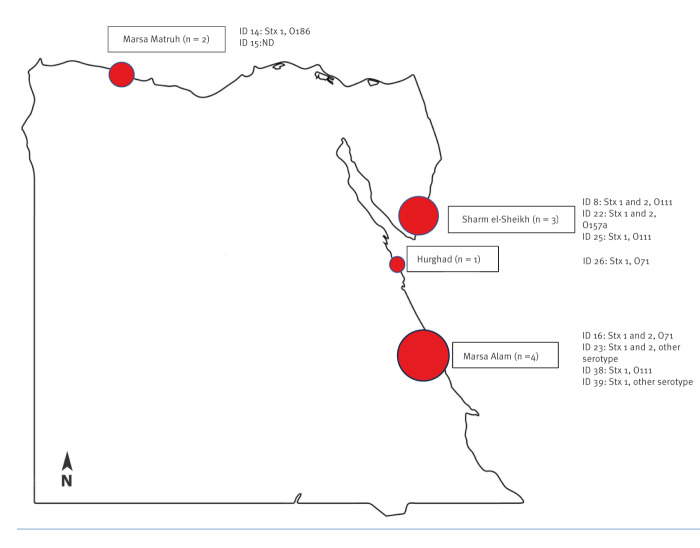
Geographic locations of holiday resorts in Egypt of children and adolescents with bloody diarrhoea and infected with Shiga toxin-producing *Escherichia coli*, northern Italy, 2023 (n  =  10)^a^

ID: identification code; ND: not determined; Other serotype: other serotype than O157, O26, O103, O111, O1O4 or O145.

^a^ Only cases considered infected in Egypt are included in the figure.

In the unexposed time, we observed 31 cases (14,783 person-days). Of the eight cases who travelled to other countries but not to Egypt, two had been in other countries after the illness and four acquired the disease weeks–months after the travel.

## Discussion

Herein, we demonstrate a statistically higher risk of STEC infection with bloody diarrhoea (thus of HUS) associated with travel to Egypt. 

The different serotypes detected from these cases exclude the possibility of a point-source outbreak. Furthermore, all children were guests at vacation resorts, thus they consumed meals and beverages provided by the facility. We could not identify any specific exposures among the cases.

The epidemiology of STEC infection in Egypt is not well documented. However, in 2014, Majowicz et al. estimated an incidence of 152.6 per 100,000 person-years in a region including Egypt compared with 47.1 per 100,000 person-years in several European countries [12]. Moreover, in 2021, Elmonir et al. detected STEC from 6.5% of raw food (milk and beef) samples in a single district in northern Egypt (Kafrelsheikh) [13]. They also demonstrated STEC from samples from cattle (12%) and humans (10%) with diarrhoea, in the same area [13]. In addition, Mervat et al. detected *E. coli* O157:H7 in 11% of patients with gastroenteritis in the Egyptian Governate of Beni Suef, with 52% of the STEC infected patients with HUS. Based on these findings, the authors highlighted the need for food control measures, water decontamination and public awareness to decrease the infections [14]. In the only available case series of HUS associated with STEC infection in Egyptian children, 132 cases were reported between 2007 and 2017 in a referral area (Mansoura University Children’s Hospital) [15] with at least 2 million children [16].

The increased risk of infections, particularly by enteric pathogens, in travellers to Egypt has been previously reported [17-24]. However, STEC infection is associated with a potentially life-threatening complication in children thus it deserves specific attention. Germany, the United Kingdom (UK) and the European Centre for Disease Prevention and Control (ECDC) have alerted of STEC infection and HUS after travel to Egypt [25-28]. Nonetheless, this had not been systematically investigated.

We have repeatedly suspected an association between HUS in children with travel abroad for vacation (particularly in Egypt). After the COVID-19 pandemic, travel has increased, in particular, to Egypt [29,30], which may have contributed to the increased numbers of STEC infections acquired in there. However, we do not have data on the number of Italians travelling to Egypt.

Besides the 10 children who became infected in Egypt, two additional patients (ID 9 and ID 30) developed initial symptoms only shortly after their return from Egypt (6–8 days after), but beyond the period we defined as exposed. Moreover, five (50%) of the 10 cases infected in Egypt developed HUS compared to 6 (18%) of the 33 cases infected elsewhere. This may be due to more virulent pathogens in Egypt. Conversely, a selection bias may be hypothesised: a certain proportion of uncomplicated infections may have been missed if resolution occurred during the vacation. In the latter case the actual risk of infection would be even greater than estimated.

Most cases, including those infected in Egypt, are clustered in the summer period. This is consistent with the well-known seasonality of STEC infections and with the increased travelling pattern of Italians during school holidays.

One of the possible limitations of our analysis is recall bias, as the information came from the retrospective interviews with the parents and clinical charts. In addition, the ItalKid-HUS Network does not cover the entire country. Nevertheless, the catchment area covers a paediatric population of 2.3 million. Furthermore, the number of cases in our study may seem small. However, STEC infection is a relatively rare condition, and the chosen method (SCCS) is statistically powerful and has been successfully used to identify associations in case series that were much smaller (e.g. 10 patients) [11].

Even though identifying the specific sources of STEC infections remains challenging, our case series highlights that travel to Egypt accounted for almost a fourth of the cases in our surveillance system in 2023. The possible vehicles of infections are many (ingestion of poorly cooked meat, dairy products, vegetables, swimming in contaminated water, direct animal contact, inter-human transmission). Nonetheless, in this study, no specific source of infection could be traced except for the general risk derived from travelling to Egypt.

## Conclusion

The risk of STEC infection related to travel to Egyptian vacation sites is worrisome, given the potential severity of the infection and the number of tourists visiting the country. Investigations to identify the sources are needed to implement preventive measures.

We urge physicians to inform families travelling to Egyptian holiday resorts with children to be very cautious and to apply all general preventive measures to avoid the infection (hand washing before meals, avoiding poorly cooked meat and vegetables, avoiding street food, avoiding direct contact with ruminants and drink bottled water).

## § Note

This designation is without prejudice to positions on status, and is in line with UNSCR 1244 and the IСJ Opinion on the Kosovo Declaration of Independence.
